# Technology matters: Co‐developing & evaluating digital support for young people with depression and anxiety, MoodHwb

**DOI:** 10.1111/camh.70004

**Published:** 2025-07-02

**Authors:** Rhys Bevan Jones

**Affiliations:** ^1^ Wolfson Centre for Young People's Mental Health & Division of Psychological Medicine and Clinical Neurosciences Cardiff University Cardiff Wales UK; ^2^ Cwm Taf Morgannwg University Health Board Rhondda Cynon Taf Wales UK

**Keywords:** Digital intervention, depression, anxiety, young people/adolescents, co‐development, bilingual

## Abstract

Depression and anxiety are common in young people, yet many do not receive adequate support. Current guidelines recommend digital interventions as an effective approach; however, there is a need for more accessible, evidence‐based programmes that are co‐developed with young people and rigorously evaluated. The bilingual digital programme, MoodHwb, was co‐designed with young people with depression and in collaboration with parents, carers, professionals and a digital media company. MoodHwb aims to support young people in managing their mood and well‐being. An early evaluation study provided initial support for the programme theory and indicated its acceptability among users. The programme has since been refined based on feedback, and a feasibility randomised controlled trial has been completed.

## Background

Depression and anxiety are common in young people and are associated with social and educational impairments, self‐harm and suicide. They can also mark the onset of long‐term mental health difficulties. Furthermore, subthreshold symptoms can affect quality of life and be precursors to disorder. However, many young people do not access or receive support, and engaging young people in prevention and early intervention programmes remains a challenge. Guidelines for the prevention and management of depression and anxiety in young people, such as those from the National Institute for Health and Care Excellence (NICE) and the American Academy of Child and Adolescent Psychiatry, emphasise the importance of providing clear information and evidence‐based psychosocial interventions for young people and families/carers. The UK NICE guidelines specifically recommend digital interventions for managing mild depression (NICE, 2019).

Digital mental health technologies—encompassing resources and interventions—offer a promising way of enhancing access to support at scale and relatively low cost. While evidence for the effectiveness of digital mental health technologies is growing, there remains a need for more programmes that are accessible, evidence‐based, developed with user input, and rigorously evaluated—to provide more options for young people when seeking support (Bevan Jones, Stallard, et al., 2020).

## The digital intervention, MoodHwb

MoodHwb is a bilingual (English and Welsh), interactive and multiplatform digital programme aimed at supporting young people to manage their mood and well‐being. It was initially co‐developed with young people aged 13–19 years for those with symptoms or a diagnosis of depression. It has since been expanded to include anxiety symptoms, as these commonly coexist. MoodHwb can be used by the young person alone, or supported by another person, such as a parent, carer or practitioner. ‘Hwb’ is the Welsh translation for ‘hub’ and can mean a ‘lift’ or ‘boost’, and HwbHwyliau is the Welsh name for the programme. Detailed information about the programme and its development process can be found in earlier publications (Bevan Jones, Thapar, Rice, et al., 2018; Bevan Jones, Thomas, Lewis, Read, & Jones, 2017).

The programme was designed to engage young people through developmentally appropriate language, illustrations, animations and interactive features. It aimed to promote self‐help, encourage help‐seeking when necessary and foster social support. The first version was founded primarily on psychoeducation, with elements of cognitive behaviour therapy (CBT), behavioural activation, positive psychology and theories from interpersonal, family systems and behaviour change perspectives. Initially developed as a website, the programme included interactive sections such as a mood diary that were also accessible via a native app for both Android and Apple operating systems.

On the welcome screen of the first version of MoodHwb, users were prompted to answer questions, for example, about their mood and anxiety levels. These responses were saved in their profile, and users were then guided to the most appropriate content via a personalised dashboard, enabling the programme to be tailored to their individual needs. In addition to the mood diary, other interactive features included a goal‐setting tool and the ability to save links to helpful resources.

Originally, an introductory section and five core sections were developed for young people covering: (1) low mood and depression, (2) possible reasons, (3) self‐help strategies, (4) sources of support and (5) related health difficulties, including anxiety. A sixth section was developed for those concerned about young people's mental health and well‐being, such as parents, carers, friends and practitioners. Each section featured a range of supportive content, including informative text, animations, personal stories, exercises and links to relevant resources.

## Original co‐development of MoodHwb

The overall framework of co‐development is illustrated in Figure S1. The process was detailed in Bevan Jones, Thapar, Rice, et al. (2018) and featured as a case study in a review on the co‐design of digital technologies (Bevan Jones, Stallard, et al., 2020). MoodHwb was developed iteratively ‘de novo’ in collaboration with young people with lived experience of depression (or deemed at high risk due to family history), as well as parents/carers and professionals from health, education, social and youth services.

During the discovery phase, initial ideas for the programme were generated through a series of 12 semi‐structured interviews. These concepts were then developed further in the codesign phase through six focus groups (three with young people, one with parents/carers, one with clinicians, one with researchers), alongside workshops with a digital media company, animator and experts in psychology/psychiatry and design. In the latter focus groups, draft designs were shown, and participants could interact with early components using tablets or laptops. The development was also informed by a systematic review of the existing literature (Bevan Jones, Thapar, Stone, et al., 2018) and by design, educational and psychological theories.

Figure S2 provides a visual account of the initial prototype's development process. The initial stages involved creating note boards and sketches informed by user and project requirements, serving as the foundation for initial design concepts discussed in the focus groups. From these discussions, wireframes—skeletal frameworks outlining each screen's layout and functionality—were developed, gradually evolving into the prototype. Collaboration with the young people, designers and an animator further refined the illustrations, character designs, scripts and animations—ensuring the prototype aligned with the intended user experience. A preliminary ‘logic model’ (programme theory) was also developed based on the qualitative work, literature review and relevant theory (Figure S3).

MoodHwb's bilingual approach aligns with NICE guidelines (2019) which recommend offering psychological therapies in the language of the young person and their family/carers when English is not their first language. This approach acknowledges the challenges in the communication of sensitive and subjective issues in another language.

## Early evaluation of the initial prototype

As part of the developmental work, an early evaluation of the initial MoodHwb prototype (Figure S1) was carried out with 44 young people (age range: 13–23 years, mean: 16.3 years) and 31 parents/carers (Bevan Jones, Thapar, et al., 2020). Participants were recruited through primary and secondary Child and Adolescent Mental Health Services (CAMHS), school counsellors and nurses, and from a previous study sample, the Early Prediction of Adolescent Depression (EPAD) study.

Participants completed questionnaires before and after using the programme, assessing the feasibility and acceptability of both the programme and evaluation process, as well as changes in mood, knowledge, attitudes and behaviour. Web usage was also monitored throughout. Additionally, a subsample of 19 young people and 12 parents/carers participated in interviews, and a focus group was held with professionals from health, education, social and youth services and charities.

Preliminary results were promising, indicating that participants found the programme acceptable. Young people gave favourable feedback on various aspects, including the overall presentation, intuitive navigation and engaging tone. They particularly appreciated the graphic elements (e.g. icons, metaphors, characters), animations, personal stories and interactive sections (e.g. mood diary). Many highlighted that the bilingual design made it more inclusive and accessible. The findings also provided initial support for the programme theory, particularly around depression literacy, self‐efficacy and reducing depressive symptoms.

## Further development and feasibility trial

A second version of MoodHwb has been developed (see Figure S4), based on feedback from the initial evaluation. Key revisions focused on enhancing navigation, audiovisual and interactive elements, personalising features according to user preferences, developing the app version, creating an additional self‐help section and expanding the CBT and other content to address both depression and anxiety symptoms. Young people have been involved throughout this development process.

A feasibility randomised controlled trial has been completed in Wales and Scotland to evaluate the acceptability and feasibility of this updated version of the programme, together with the trial methods—following guidance for complex interventions. Full details of the trial protocol are provided in Bevan Jones et al. (2023).

During a pretrial acceptability phase, young people in Wales and Scotland were granted access to the new prototype. Recruitment was conducted through school counsellors, charities, youth advisory groups and self‐referrals. Participants were interviewed to assess whether the changes were acceptable ahead of the trial and to examine the programme's transferability beyond Wales. Additionally, practitioners from health, education and youth services provided further feedback on the updated prototype.

The feasibility trial compared MoodHwb plus usual care with a digital information pack plus usual care (Figure S5). Young people aged 13–19 years with depressive symptoms, along with their parents/carers, were recruited via schools, mental health services, youth organisations, charities and voluntary self‐referrals. Data analysis is currently in progress, focusing on the main outcomes: the feasibility and acceptability of MoodHwb, including usage, design and content, as well as the trial methods, including recruitment and retention rates, assessed 2 months post‐randomisation. Secondary outcomes, also measured at 2 months post‐randomisation, include potential impact on mental health knowledge and stigma, help‐seeking behaviour, well‐being and symptoms of depression and anxiety.

## Conclusions

MoodHwb is a bilingual digital programme that aims to support young people with their mood and well‐being, especially those experiencing elevated depressive and anxiety symptoms. It also helps families, carers, friends and professionals involved in supporting these young people. Available both as a website and an app, MoodHwb can be used independently or be integrated alongside clinical care, for example, in counselling or therapy sessions—offering flexibility in its application.

MoodHwb is currently available only in the research setting, and the next steps will be guided by the results from the feasibility trial and may include further evaluation and implementation work in the United Kingdom and internationally. Designed with an emphasis on inclusivity—reflected in its bilingual approach using universally appealing design (e.g. icons, characters)—it is well‐positioned for translation and adaptation in other countries where multiple languages are widely spoken.

Digital mental health technologies such as MoodHwb have the potential to expand access to effective therapies at a relatively low cost, addressing the need for accessible, evidence‐based technologies developed with user input and evaluated rigorously. The articles published on MoodHwb's development and evaluation, along with the broader review and guidance on co‐design, have also helped to inform others involved in digital mental health research.

## Conflict of interest statement

The author has declared that he has no competing or potential conflicts of interest.

## Funding information

The work was supported by the National Institute for Health Research (NIHR) and Health and Care Research Wales (HCRW) Doctoral and Postdoctoral Fellowship programmes, grant numbers NIHR‐FS‐2012 and NIHR‐PDF‐2018. The work was also supported by the National Centre for Mental Health and the Wolfson Centre for Young People's Mental Health (established with support from the Wolfson Foundation).

## Ethical information

No ethical approval was required for this article. Details of the approvals for each stage in the development/evaluation of MoodHwb can be found in the respective articles.

## Supporting information


**Figure S1.** General framework for the development of the digital technology MoodHwb (from Bevan Jones, Stallard, et al., 2020).
**Figure S2.** Development of welcome screen and user flow of MoodHwb: notes/sketches (above), wireframes (centre), early designs (below) (from Bevan Jones, Stallard, et al., 2020).
**Figure S3.** Logic model for MoodHwb (above), including potential pathways (below) (from Bevan Jones et al., 2023).
**Figure S4.** MoodHwb (v2) welcome screen (main image/right) and open menu (left).
**Figure S5.** Participant flow diagram. NB: Web/app usage data of those in the intervention arm will be collected for 6 months after baseline (from Bevan Jones et al., 2023).

## Data Availability

Data availability does not apply for this article. Details of the availability for each stage in the development/evaluation of MoodHwb can be found in the respective articles.

## References

[camh70004-bib-0001] Bevan Jones, R. , Merry, S. , Stallard, P. , Randell, E. , Weavers, B. , Gray, A. , … & Simpson, S.A. (2023). Further development and feasibility randomised controlled trial of a digital programme for adolescent depression, MoodHwb: Study protocol. BMJ Open, 13, e070369. 10.1136/bmjopen-2022-070369 PMC10254867 37277220

[camh70004-bib-0002] Bevan Jones, R. , Stallard, P. , Agha, S.S. , Rice, S. , Werner‐Seidler, A. , Stasiak, K. , … & Merry, S. (2020). Practitioner review: Co‐design of digital mental health technologies with children and young people. Journal of Child Psychology and Psychiatry, 61(8), 928–940. 32572961 10.1111/jcpp.13258 PMC7611975

[camh70004-bib-0003] Bevan Jones, R. , Thapar, A. , Rice, F. , Beeching, H. , Cichosz, R. , Mars, B. , … & Simpson, S.A. (2018). A web‐based psychoeducational intervention for adolescent depression: Design and development of MoodHwb. JMIR Mental Health, 5(1), e13. 29449202 10.2196/mental.8894 PMC5832901

[camh70004-bib-0004] Bevan Jones, R. , Thapar, A. , Rice, F. , Mars, B. , Agha, S.S. , Smith, D.J. , … & Simpson, S.A. (2020). A digital intervention for adolescent depression (MoodHwb): Mixed‐methods feasibility evaluation. JMIR Mental Health. 7(7), e14536. 32384053 10.2196/14536 PMC7395255

[camh70004-bib-0005] Bevan Jones, R. , Thapar, A. , Stone, Z. , Thapar, A.K. , Jones, I. , Smith, D. , & Simpson, S.A. (2018). Psychoeducational interventions in adolescent depression: A systematic review. Patient Education and Counseling, 101(5), 804–816. 29103882 10.1016/j.pec.2017.10.015 PMC5933524

[camh70004-bib-0006] Bevan Jones, R. , Thomas, J. , Lewis, J. , Read, S. , & Jones, I. (2017). Translation: From bench to brain – Using the visual arts and metaphors to engage and educate. Research for All, 1(2), 265–283.

[camh70004-bib-0007] National Institute for Health and Clinical Excellence (NICE) . (2019). Depression in children and young people. *NICE clinical guideline* . Available from: http://guidance.nice.org.uk/CG28/Guidance 31577402

