# Supplementary material for: Technology matters: Co‐developing & evaluating digital support for young people with depression and anxiety, MoodHwb
Source: Child Adolesc Ment Health. 2025 Jul 2;31(2):159–61. doi: 10.1111/camh.70004 (PMC13116025; doi:10.1111/camh.70004)
Supplement: Supplementary file 1 — Figure S1. General framework for the development of the digital technology MoodHwb (from Bevan Jones, Stallard, et al., 2020). Figure S2. Development of welcome screen and user flow of MoodHwb: notes/sketches (above), wireframes (centre), early designs (below) (from Bevan Jones, Stallard, et al., 2020). Figure S3. Logic model for MoodHwb (above), including potential pathways (below) (from Bevan Jones et al., 2023). Figure S4. MoodHwb (v2) welcome screen (main image/right) and open menu (left). Figure S5. Participant flow diagram. NB: Web/app usage data of those in the intervention arm will be collected for 6 months after baseline (from Bevan Jones et al., 2023). [file CAMH-31-159-s001.docx]

**Supporting Information**

**Technology matters: Co-developing & evaluating digital support for young people with depression and anxiety, MoodHwb**


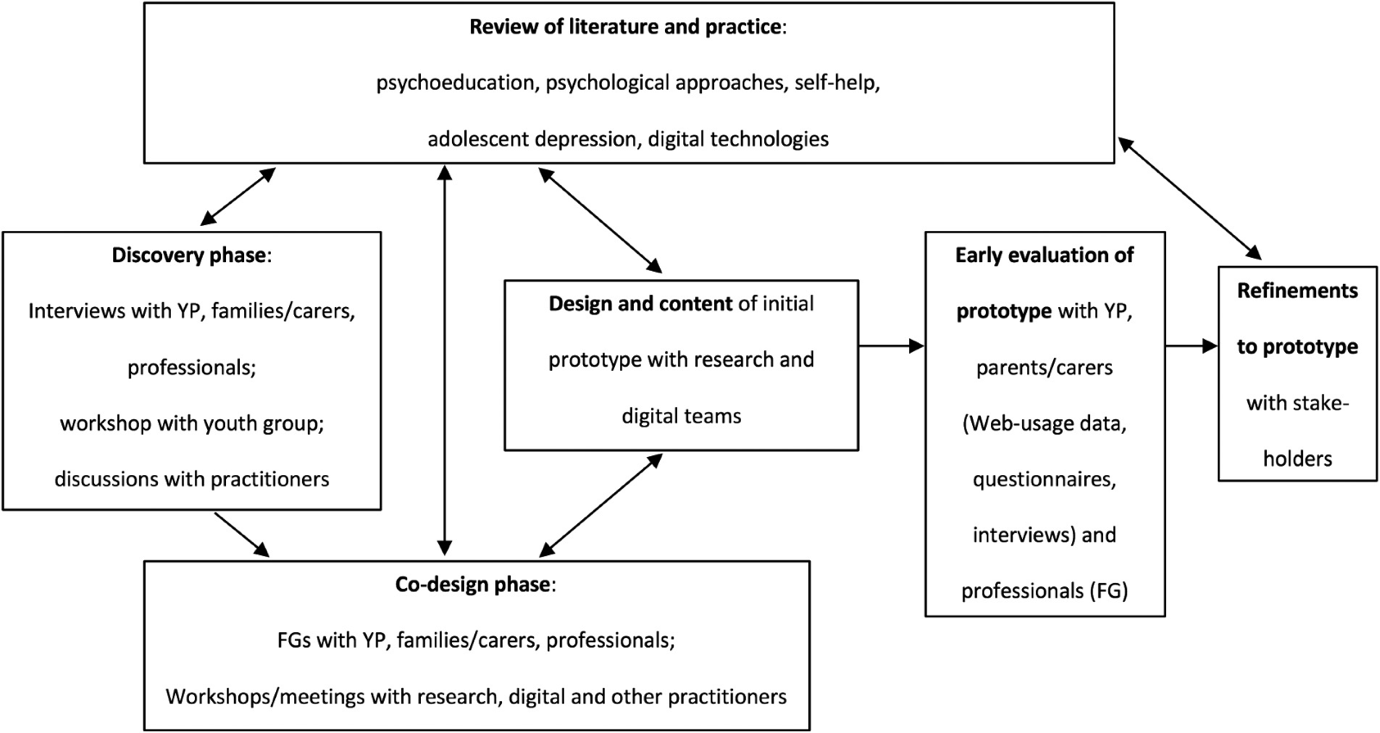


**Figure S1:** General framework for the development of the digital technology MoodHwb (from Bevan Jones et al., 2020)


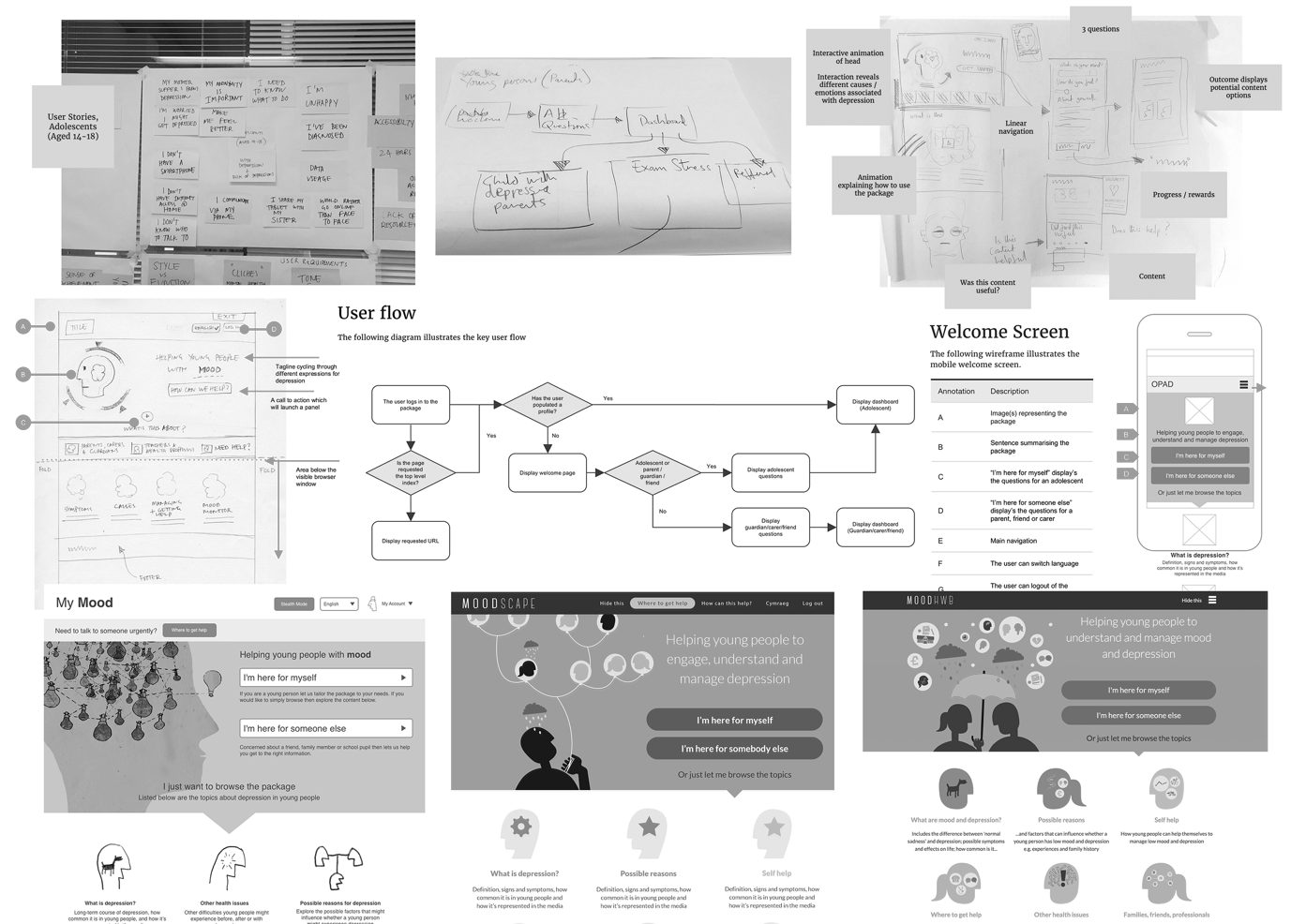


**Figure S2:** Development of welcome screen and user-flow of MoodHwb: notes/sketches (above), wireframes (centre), early designs (below) (from Bevan Jones et al., 2020)


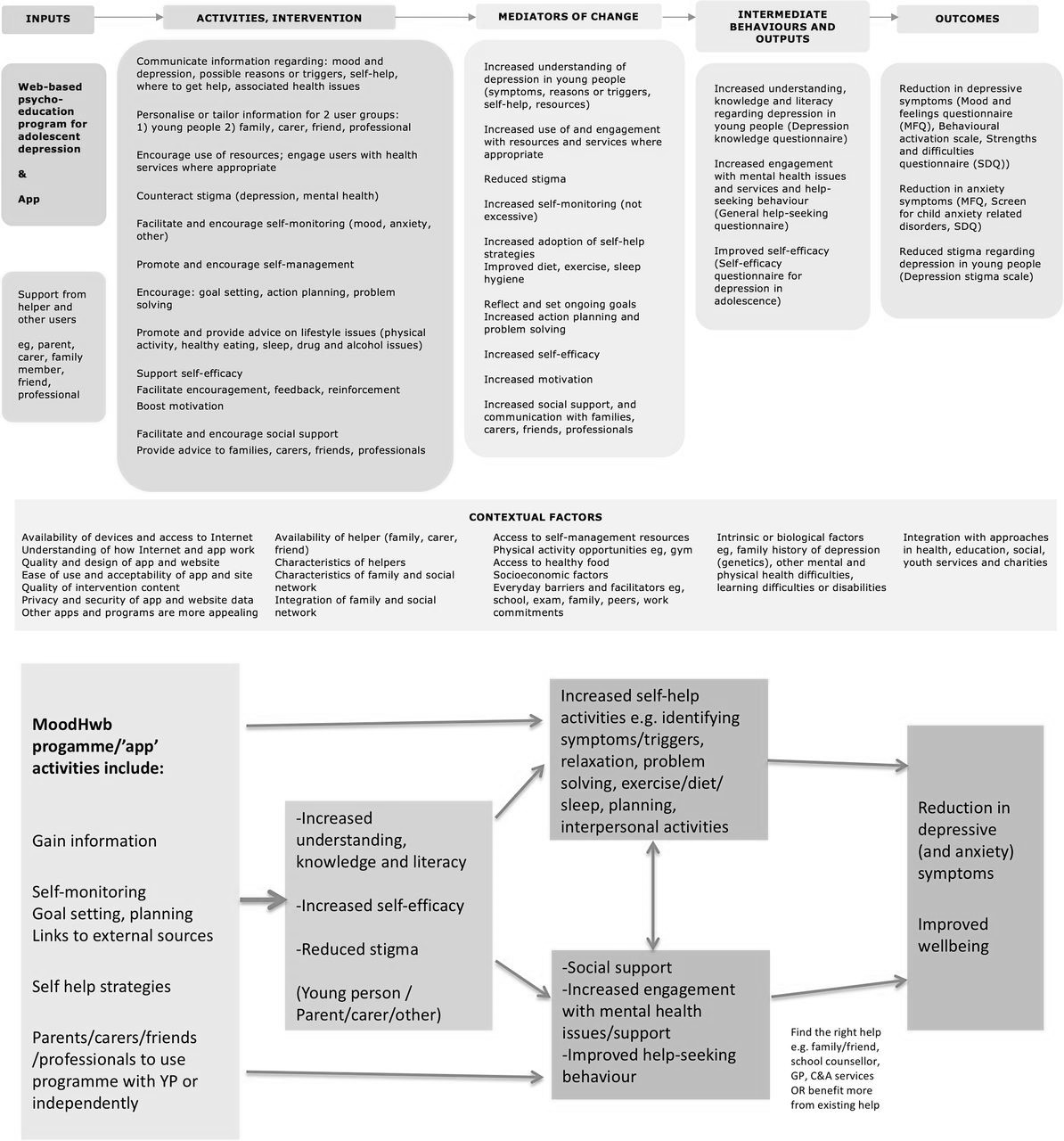


**Figure S3:** Logic model for MoodHwb (above), including potential pathways (below) (from Bevan Jones et al., 2023).


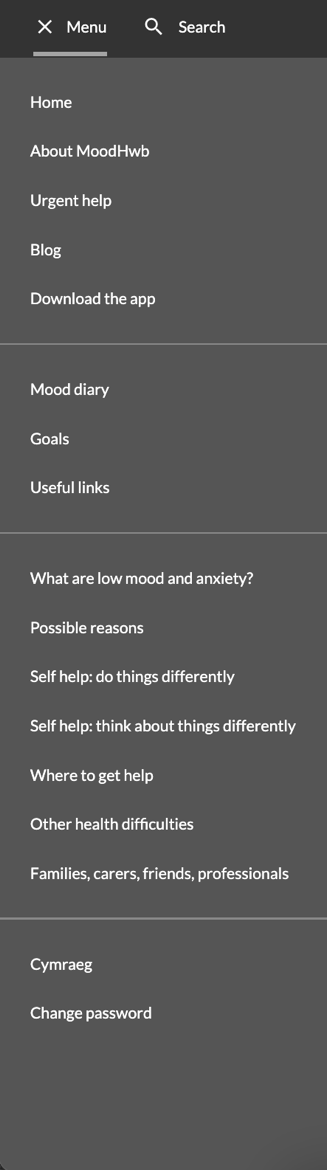

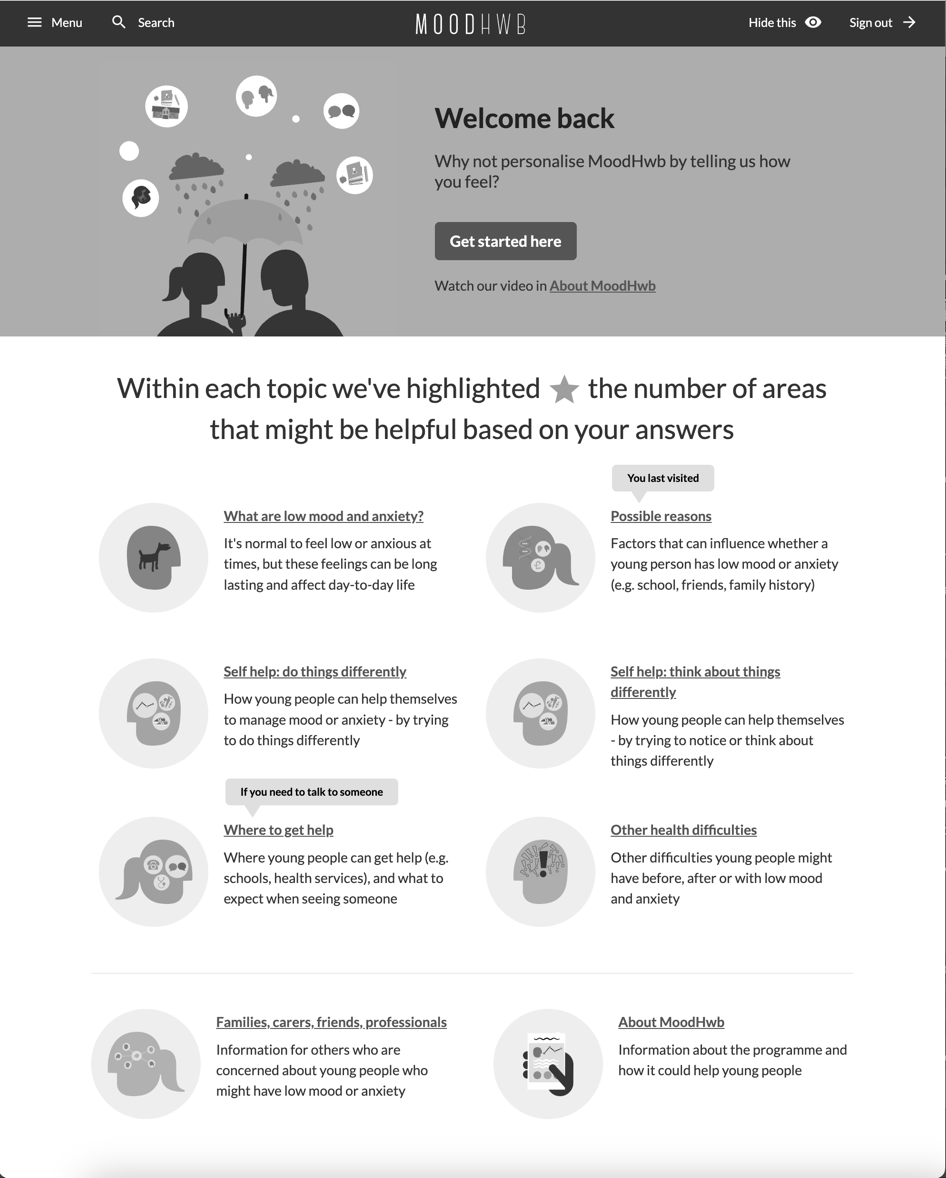


**Figure S4**: MoodHwb (v2) welcome screen (main image/right) and open menu (left)


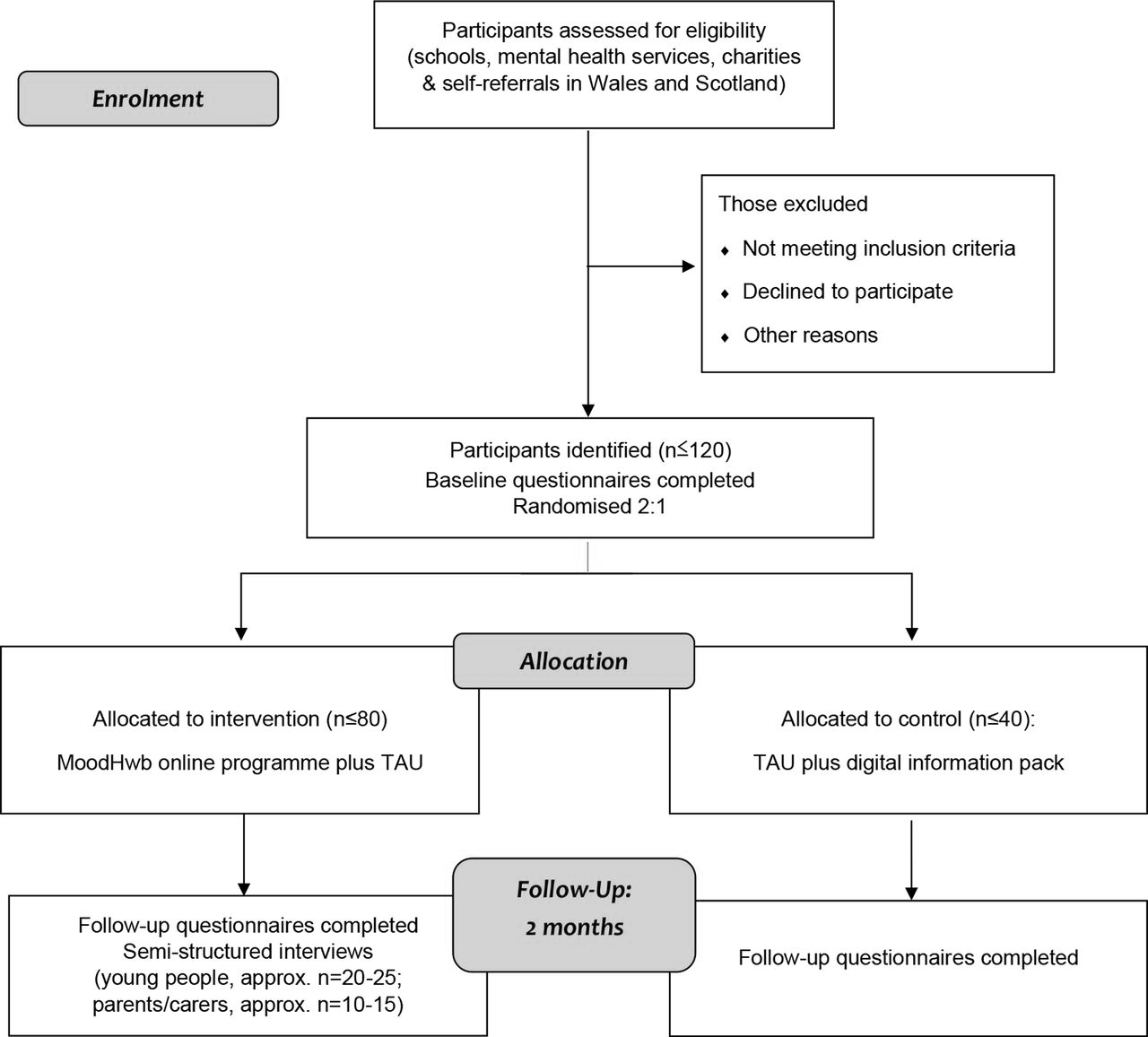


**Figure S5:** Participant flow diagram. NB: Web/app usage data of those in the intervention arm will be collected for 6 months after baseline (from Bevan Jones et al., 2023).
